#  Concentric and Eccentric: Muscle Contraction or Exercise? 

**DOI:** 10.2478/hukin-2013-0019

**Published:** 2013-07-05

**Authors:** Johnny Padulo, Guillaume Laffaye, Luca Paolo Ardigò, Karim Chamari

**Affiliations:** 1 Faculty of Medicine and Surgery, University of “Tor Vergata” Rome (Italy).; 2 SPO Sport Performance Optimization Lab, CNMSS, Tunis (Tunisia); 3 UR CIAMS – Motor Control and Perception Group, Sport Sciences Department, Université Paris-Sud, Orsay (France).; 4 School of Exercise and Sport Science, University of Verona (Italy).; 5 Orthopaedic and Sports Medicine Hospital, Doha (Qatar).

 This inclusion considers the use and possible misuse of the terms “Concentric and Eccentric” in three possible contexts: first, the origin of terms; second, different approaches; and third, the possible uses. To the best of our knowledge, three articles (Aboodarda, 2011;  Bdel-Aziem and Mohammad, 2012  ;  Krol and Mynarski, 2012  ) have been published in the *Journal of Human Kinetics * misusing the term “concentric/eccentric exercise” while none of the articles have used the terms correctly. The purpose of this letter is to foster the use of the terminology ‘positive/negative work’ together with ‘concentric/eccentric contraction’ to ease references search (i.e., through key words) and comprehension. 

##  When did these terms initially appear and what do they mean? 

 The origin of the terms “Concentric and Eccentric” is related to muscle contraction in basic physiology science. Back to 1925, Hill defined two types of muscle contractions (  Hill, 1925  ): isometric where muscle length does not change during contraction and isotonic where tension remains unchanged or could also vary while the muscle’s length changes. There are two types of isotonic contractions: (a) concentric and (b) eccentric (  Hill, 1925  ). In a concentric contraction, the muscle tension rises to meet the resistance, then remains stable as the muscle shortens. During eccentric contraction, the muscle lengthens as the resistance is greater than the force the muscle is producing. 

##  Which areas do the terms concentric/eccentric cover? 

 In the following years, the terms “Concentric and Eccentric” were frequently used in scientific manuscripts in different areas: physiology, biomechanics, and neuromechanics. In * PubMed * a search concerning the years between 1975 and 2012, found * n * =190087 articles using the words “muscle contraction” vs. * n * =2302/1582 articles with “eccentric/concentric exercises”. Several authors have misused the term “concentric/eccentric work or exercise” for an exercise with displacement of the body upwards to overcome gravity (positive work) or landing (negative work), whereas the terms “Eccentric/Concentric” are strictly linked to a muscular behavior. Thus, we believe that the discussed terms cannot be used in all contexts. 

##  Is it judicious to use (only) Eccentric/concentric for exercises? 

 From the point of view of physics, during positive work (rising/accelerating) or negative work (lowering/decelerating) (  Asmussen, 1953 ) some muscles are in eccentric mode. For instance, during concentric elbow flexion, the biceps brachii contracts concentrically, whereas the antagonist muscle, the triceps brachii, contracts mildly eccentrically – to allow movement precision and control. Another example occurs in the leg press machine, during resisting the quadriceps contracts eccentrically, whereas the biceps femoris contracts mildly concentrically – to allow movement precision and to control the tension of the extensors that could, if applied without an antagonist, overstress the knee joint and even damage the ACL (anterior cruciate ligament). In both cases, a necessary dynamical description of the exercise – e.g. ‘there is positive/negative work’ – is missing. In the first example, it should be underlined that positive work is developed, while the second example features negative work. 

 Furthermore, the use of these terms in both exercise and muscle contraction has created confusion (  Faulkner, 2003  ). Considering the need to clarify this question we propose that ”positive or negative work” (Bosco, 1982) terms are more appropriate for describing some exercise while in another context it would be more correct to use ”flexion – extension” or ”adduction – abduction” for single joint exercise or “traction or pushed per multi-joint exercise” for instance (  Zatsiorsky and Prilutsky, 2012  ). 

 The correct use of terms “Eccentric and concentric” can be valuable for understanding results in a journal article and deciding whether the authors’ conclusions are justified by the data. To avoid confusion, words such as positive (concentric) or negative (eccentric) exercise are preferable as they indicate the importance of the outcome. 

 We believe Sports Science still presents some confusion for some other concepts and we invite all our colleagues to discuss them in letters to the editors as we did in this short letter. 

## References

[b1-jhk-37-5]  Aboodarda   SJ ,  Shariff   MA ,  Muhamed   AM ,  Ibrahim   F ,  Yusof   A  ( 2011 ). Electromyographic activity and applied load during high intensity elastic
resistance and nautilus machine exercises. J Hum Kinet.

[b2-jhk-37-5]  Asmussen   E  ( 1953 ). Positive and negative muscular work. Acta Physiol Scand.

[b3-jhk-37-5]  Bdel-Aziem   AA ,  Mohammad   WS  ( 2012 ). Plantar-flexor Static Stretch Training Effect on Eccentric and Concentric
Peak Torque - A comparative Study of Trained versus Untrained Subjects. J Hum Kinet.

[b4-jhk-37-5]  Bosco   C ,  Viitasalo   JT ,  Komi   PV ,  Luhtanen   P  ( 1982 ). Combined effect of elastic energy and myoelectrical potentiation during
stretch-shortening cycle exercise. Acta Physiol Scand.

[b5-jhk-37-5]  Faulkner   JA  ( 2003 ). Terminology for contractions of muscles during shortening, while
isometric, and during lengthening. J Appl Physiol.

[b6-jhk-37-5]  Hill   AV  ( 1925 ). Length of muscle, and the heat and tension developed in an isometric
contraction. J Physiol.

[b7-jhk-37-5]  Krol   H ,  Mynarski   W  ( 2012 ). A comparison of mechanical parameters between the counter movement jump
and drop jump in biathletes. J Hum Kinet.

[b8-jhk-37-5]  Zatsiorsky   VM ,  Prilutsky   BI  ( 2012 ). Biomechanics of Skeletal Muscles. Human Kinetics.

